# Supplementary material for: Patterns of Intron Gain and Loss in Fungi
Source: PLoS Biol. 2004 Nov 30;2(12):e422. doi: 10.1371/journal.pbio.0020422 (PMC532390; doi:10.1371/journal.pbio.0020422)
Supplement: Table S1 — Also available at http://genes.mit.edu/NielsenEtAl/. (4.3 MB ZIP). [file pbio.0020422.st001.zip › NielsenEtAl/html/153.html]

AN1229.1.NCU02706.1.MG04454.1.FG01020.1


```
 CLUSTAL W (1.82) Multiple Sequence Alignments - Introns Inserted


Sequence 1: MG04454.1	227 aa
Sequence 2: FG01020.1	218 aa
Sequence 3: NCU02706.1	227 aa
Sequence 4: AN1229.1	183 aa
Alignment Length: 229 aa
Number Identitical Residues: 64 aa
Alignment Score (without introns) 4281


MG04454.1 	-MAS-SGAGGWSTLRNQARTLEKD~TEALFHTYSQ~IATSSNIPAKPTEQERETEARLRE
NCU02706.1	MAATTSSTQGWAQLRQQARSLETQ0TEMLLHTYAQ~FSNQTNIPPKPTEEERHTETRLRE
FG01020.1 	-MSTPSGTGSWTSLIQQARQLEK-~---------Q0FAAAPNIPPKPTEKERETEKKLEE
AN1229.1  	-MASSTGAG-WAQLRQQARSLETQ0TESLFHTYSQ~YASMTKLPPQPAEEEIRNEHQIRD
          	  ::.:.:  *: * :*** **.. :.    : :*  :  .::*.:*:*:* ..* ::.:

MG04454.1 	ILDK0RENVIAQLTRLLDSDSDLASSSTKVRNLALFRDKLAEQRRELPRLRSRIQETRNR
NCU02706.1	LLDK0RESVISHLTRLLTSDP--SPSALKQNNLSLLRDKLSDHRRDLSRLRSTLESARTR
FG01020.1 	LLEK0REDVISKLVRLLDSEAALTSSAMKQNNLALFQKKLASHRKDLIRLRSNLQEARNR
AN1229.1  	LLEK0RESLLAQLARLLDSEATLTSSALKQNNLARHREVLAEHKRELSRLTAAIAELRDR
          	:*:* **.::::*.*** *:.  :.*: * .**:  :. *:.::::* ** : : . * *

MG04454.1 	ANLLSNVRNDIDAYRASNPEQAEAEYMLDERSRIDNSHSMADDVLYQA~YAINDNFNSQR
NCU02706.1	ANLLGSVRDDISAYRAANPEQGEADYMLDERGRLDRSHDVADSVLSQA~YAVQDSFASQR
FG01020.1 	ANLLTNVRSDIDQYRQNNPEAAEADYMLQERNRIDNSNSMADNVLSQA~YAVNDNFNLQR
AN1229.1  	ANLLSNVRSDINAYRSSNPAAAESEYMLEERRRIDESHGVIDGVLSQA2-----------
          	**** .**.**. **  **  .*::***:** *:*.*:.: *.** **            

MG04454.1 	EMLASINRRITLAASQVPGINTLMTRISAKKRRDGIIMGSFIAICFFVFWFFL
NCU02706.1	ERLAHINRRITLAASQVPGINTLITRISARKRRDGIIMGSFIAFCFLMFWFFS
FG01020.1 	ETLASINRRITHAASQVPGINTLIGRISAKKRRDGIIMGGFVAFCFIVFFLFS
AN1229.1  	---ARHFRALTGGLWALP----------AKSRG--------------------
          	   *   * :* .   :*          *:.*
```
